# Benzyl *N*′-benzhydrylidene­hydrazine­carbodithio­ate

**DOI:** 10.1107/S1600536808039408

**Published:** 2008-11-29

**Authors:** Bing-Xiang Zhang

**Affiliations:** a Department of Chemistry, Taishan University, 271021 Taian, Shandong, People’s Republic of China

## Abstract

In the title mol­ecule, C_21_H_18_N_2_S_2_, the C=N—N angle of 117.6 (2)° is significantly smaller than the ideal value of 120° expected for *sp* ^2^-hybridized N atoms. This is probably a consequence of repulsion between the nitro­gen lone pairs and the adjacent N atom, as suggested in Zheng, Qiu, Lin & Liu [*Acta Cryst.* (2006), E**62**, o1913–o1914]. The two neighbouring benzene rings form a dihedral angle of 75.95 (3)° with each other, while subtending dihedral angles of 84.18 (3) and 8.44 (2)° with the third ring in the structure.

## Related literature

For related literature on ligands derived from *S*-benzyl­dithio­carbazate (SBDTC), see: Ali *et al.* (2002 ▶, 2008 ▶); Crouse *et al.* (2004 ▶); Tarafder *et al.* (2001 ▶, 2008 ▶); Zheng *et al.* (2006 ▶). For bond-length data, see: Allen *et al.* (1987 ▶).
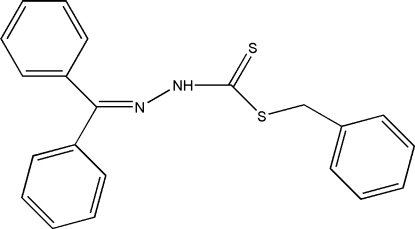


## Experimental

### 

#### Crystal data

C_21_H_18_N_2_S_2_
*M* *_r_* = 362.49
Monoclinic, 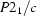

*a* = 20.2903 (14) Å
*b* = 9.0951 (6) Å
*c* = 10.5818 (7) Å
β = 103.9240 (10)°
*V* = 1895.4 (2) Å^3^
*Z* = 4
Mo *K*α radiation
μ = 0.29 mm^−1^
*T* = 295 (2) K
0.12 × 0.10 × 0.06 mm

#### Data collection

Bruker APEX2 CCD area-detector diffractometer
Absorption correction: multi-scan (*SADABS*; Bruker, 2005 ▶) *T*_min_ = 0.967, *T*_max_ = 0.983
9776 measured reflections
3363 independent reflections
2191 reflections with *I* > 2σ(*I*)
*R*_int_ = 0.039

#### Refinement

*R*[*F* ^2^ > 2σ(*F* ^2^)] = 0.047
*wR*(*F* ^2^) = 0.116
*S* = 1.05
3363 reflections
214 parameters
H-atom parameters constrained
Δρ_max_ = 0.22 e Å^−3^
Δρ_min_ = −0.23 e Å^−3^

### 

Data collection: *APEX2* (Bruker, 2005 ▶); cell refinement: *SAINT* (Bruker, 2005 ▶); data reduction: *SAINT*; program(s) used to solve structure: *SHELXS97* (Sheldrick, 2008 ▶); program(s) used to refine structure: *SHELXL97* (Sheldrick, 2008 ▶); molecular graphics: *SHELXTL* (Sheldrick, 2008 ▶); software used to prepare material for publication: *SHELXTL*.

## Supplementary Material

Crystal structure: contains datablocks global, I. DOI: 10.1107/S1600536808039408/bg2222sup1.cif

Structure factors: contains datablocks I. DOI: 10.1107/S1600536808039408/bg2222Isup2.hkl

Additional supplementary materials:  crystallographic information; 3D view; checkCIF report

## Figures and Tables

**Fig. 1.**
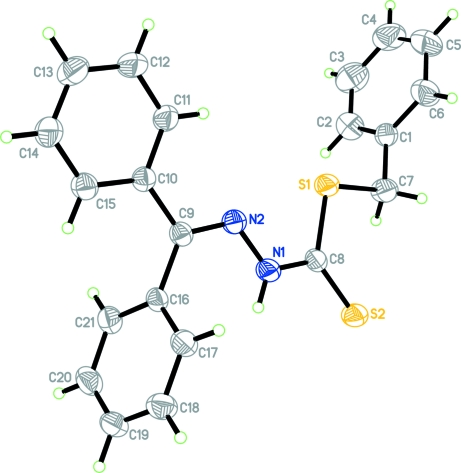
The molecular structure of (I), with displacement ellipsoids drawn at the 30% probability level.

## supplementary crystallographic information

### Comment

In recent years, the interesting coordination chemistry and increasingly relevant biomedical properties of ligands derived from *S*-benzyldithiocarbazate(SBDTC) have received much attention (Ali *et al.*, 2002, 2008; Crouse *et al.*, 2004; Tarafder *et al.*, 2001, 2008). In order to search for new ligands derived from SBDTC, the title compound C_21_H_18_N_2_S_2_ (I) was synthesized and its crystal structure determined. Fig 1 shows a molecular diagram of (I), where bond lengths and angles are basically in normal ranges (Allen *et al.*, 1987). The C=N bond length of 1.293 (3) Å(C9=N2) shows double-bond character. The C=N—N angle of 117.6 (2) ° is significantly smaller than the ideal value of 120 ° expected for *sp*^2^-hybridized N atoms. This is probably a consequence of repulsion between the nitrogen lone pairs and the adjacent N atom (Zheng *et al.*, 2006). The C10—C15, C16—C21 benzene rings are oriented at 84.18 (3) °, 8.44 (2) ° with respect to the C1—C6 one. The dihedral angle formed by the C10—C15 and C16—C21 rings is 75.95 (3) °.

### Experimental

The title compound was synthesized by the reaction of Hydrazinecarbodithioic acid benzyl ester(1 mmol, 198.3 mg) with Diphenyl-methanone(1 mmol, 182.2 mg) in ethanol(15 ml) under reflux conditions (338 K) for 5 h. The solvent was removed and the solid product recrystallized from tetrahydrofuran. After six days yellow crystals suitable for X-ray diffraction study were obtained.

### Refinement

All H atoms were placed in idealized positions (C—H = 0.93— 0.97 Å, N—H = 0.86 Å) and refined as riding atoms. For those bound to C, *U*_iso_(H) = 1.2 or 1.5*U*_eq_(C). while for those bound to N, *U*_iso_(H) = 1.2 *U*_eq_(N).

### Crystal data

**Table:**

C_21_H_18_N_2_S_2_	*F*_000_ = 760
*M**_r_* = 362.49	*D*_x_ = 1.270 Mg m^−^^3^
Monoclinic, *P*2_1_/*c*	Mo *K*α radiation λ = 0.71073 Å
Hall symbol: -P 2ybc	Cell parameters from 1615 reflections
*a* = 20.2903 (14) Å	θ = 3.0–22.7º
*b* = 9.0951 (6) Å	µ = 0.29 mm^−^^1^
*c* = 10.5818 (7) Å	*T* = 295 (2) K
β = 103.9240 (10)º	Block, yellow
*V* = 1895.4 (2) Å^3^	0.12 × 0.10 × 0.06 mm
*Z* = 4	

### Data collection

**Table:**

Bruker APEX2 CCD area-detector diffractometer	3363 independent reflections
Radiation source: fine-focus sealed tube	2191 reflections with *I* > 2σ(*I*)
Monochromator: graphite	*R*_int_ = 0.039
*T* = 295(2) K	θ_max_ = 25.1º
φ and ω scans	θ_min_ = 2.1º
Absorption correction: multi-scan(SADABS; Bruker, 2005)	*h* = −24→24
*T*_min_ = 0.967, *T*_max_ = 0.983	*k* = −10→10
9776 measured reflections	*l* = −11→12

### Refinement

**Table:**

Refinement on *F*^2^	Secondary atom site location: difference Fourier map
Least-squares matrix: full	Hydrogen site location: inferred from neighbouring sites
*R*[*F*^2^ > 2σ(*F*^2^)] = 0.047	H-atom parameters constrained
*wR*(*F*^2^) = 0.116	*w* = 1/[σ^2^(*F*_o_^2^) + (0.0462*P*)^2^ + 0.2919*P*] where *P* = (*F*_o_^2^ + 2*F*_c_^2^)/3
*S* = 1.05	(Δ/σ)_max_ = 0.001
3363 reflections	Δρ_max_ = 0.22 e Å^−^^3^
214 parameters	Δρ_min_ = −0.23 e Å^−^^3^
Primary atom site location: structure-invariant direct methods	Extinction correction: none

### Special details

**Table:**

Geometry. All e.s.d.'s (except the e.s.d. in the dihedral angle between two l.s. planes) are estimated using the full covariance matrix. The cell e.s.d.'s are taken into account individually in the estimation of e.s.d.'s in distances, angles and torsion angles; correlations between e.s.d.'s in cell parameters are only used when they are defined by crystal symmetry. An approximate (isotropic) treatment of cell e.s.d.'s is used for estimating e.s.d.'s involving l.s. planes.
Refinement. Refinement of *F*^2^ against ALL reflections. The weighted *R*-factor *wR* and goodness of fit *S* are based on *F*^2^, conventional *R*-factors *R* are based on *F*, with *F* set to zero for negative *F*^2^. The threshold expression of *F*^2^ > σ(*F*^2^) is used only for calculating *R*-factors(gt) *etc*. and is not relevant to the choice of reflections for refinement. *R*-factors based on *F*^2^ are statistically about twice as large as those based on *F*, and *R*- factors based on ALL data will be even larger.

### Fractional atomic coordinates and isotropic or equivalent isotropic displacement parameters (Å^2^)

**Table:**

	*x*	*y*	*z*	*U*_iso_*/*U*_eq_	
S1	0.29194 (4)	0.73862 (8)	0.23650 (8)	0.0593 (2)	
S2	0.15831 (4)	0.59677 (8)	0.10325 (8)	0.0636 (3)	
N1	0.17341 (11)	0.8548 (2)	0.2139 (2)	0.0539 (6)	
H1	0.1299	0.8630	0.1909	0.065*	
N2	0.21181 (11)	0.9673 (2)	0.2809 (2)	0.0508 (6)	
C1	0.39011 (8)	0.5783 (2)	0.1752 (2)	0.0578 (8)	
C2	0.41212 (13)	0.6495 (2)	0.0766 (2)	0.0836 (10)	
H2	0.3807	0.6909	0.0070	0.100*	
C3	0.48112 (15)	0.6589 (3)	0.0820 (3)	0.1024 (13)	
H3	0.4958	0.7065	0.0160	0.123*	
C4	0.52811 (9)	0.5971 (3)	0.1861 (3)	0.1042 (14)	
H4	0.5743	0.6034	0.1897	0.125*	
C5	0.50611 (11)	0.5259 (3)	0.2847 (3)	0.1084 (14)	
H5	0.5376	0.4845	0.3543	0.130*	
C6	0.43711 (12)	0.5165 (3)	0.2793 (2)	0.0868 (11)	
H6	0.4224	0.4689	0.3452	0.104*	
C7	0.31560 (14)	0.5689 (3)	0.1698 (4)	0.0709 (9)	
H7A	0.3065	0.4857	0.2204	0.085*	
H7B	0.2898	0.5566	0.0805	0.085*	
C8	0.20349 (13)	0.7318 (3)	0.1840 (2)	0.0475 (7)	
C9	0.18138 (13)	1.0901 (3)	0.2909 (2)	0.0450 (6)	
C10	0.22361 (13)	1.2075 (3)	0.3651 (2)	0.0449 (6)	
C11	0.29263 (14)	1.1870 (3)	0.4165 (3)	0.0603 (8)	
H11	0.3129	1.0997	0.4000	0.072*	
C12	0.33158 (15)	1.2931 (3)	0.4912 (3)	0.0714 (9)	
H12	0.3777	1.2770	0.5256	0.086*	
C13	0.30214 (17)	1.4240 (3)	0.5153 (3)	0.0718 (9)	
H13	0.3284	1.4959	0.5664	0.086*	
C14	0.23479 (16)	1.4474 (3)	0.4641 (3)	0.0664 (8)	
H14	0.2153	1.5362	0.4792	0.080*	
C15	0.19518 (15)	1.3404 (3)	0.3898 (3)	0.0554 (7)	
H15	0.1491	1.3574	0.3559	0.067*	
C16	0.10755 (13)	1.1135 (3)	0.2328 (2)	0.0437 (6)	
C17	0.05888 (14)	1.0439 (3)	0.2829 (3)	0.0564 (7)	
H17	0.0723	0.9829	0.3550	0.068*	
C18	−0.00925 (15)	1.0638 (3)	0.2275 (3)	0.0653 (8)	
H18	−0.0414	1.0176	0.2634	0.078*	
C19	−0.02962 (16)	1.1503 (3)	0.1210 (3)	0.0664 (9)	
H19	−0.0757	1.1632	0.0839	0.080*	
C20	0.01760 (16)	1.2191 (3)	0.0678 (3)	0.0670 (8)	
H20	0.0035	1.2776	−0.0058	0.080*	
C21	0.08623 (15)	1.2016 (3)	0.1237 (3)	0.0569 (7)	
H21	0.1181	1.2491	0.0880	0.068*	

### Atomic displacement parameters (Å^2^)

**Table:**

	*U*^11^	*U*^22^	*U*^33^	*U*^12^	*U*^13^	*U*^23^
S1	0.0445 (4)	0.0491 (4)	0.0788 (6)	0.0040 (3)	0.0042 (4)	−0.0127 (4)
S2	0.0521 (5)	0.0564 (5)	0.0746 (6)	−0.0006 (4)	0.0000 (4)	−0.0137 (4)
N1	0.0414 (13)	0.0488 (13)	0.0692 (16)	0.0060 (11)	0.0091 (12)	−0.0080 (12)
N2	0.0458 (14)	0.0466 (13)	0.0590 (15)	0.0028 (11)	0.0109 (11)	−0.0066 (12)
C1	0.0502 (18)	0.0447 (16)	0.075 (2)	0.0061 (14)	0.0092 (16)	−0.0101 (16)
C2	0.079 (3)	0.085 (2)	0.083 (3)	0.004 (2)	0.012 (2)	0.005 (2)
C3	0.092 (3)	0.112 (3)	0.113 (3)	−0.017 (3)	0.043 (3)	−0.008 (3)
C4	0.056 (2)	0.103 (3)	0.157 (4)	−0.003 (2)	0.032 (3)	−0.015 (3)
C5	0.055 (2)	0.116 (3)	0.142 (4)	0.008 (2)	0.000 (2)	0.022 (3)
C6	0.057 (2)	0.096 (3)	0.105 (3)	0.004 (2)	0.013 (2)	0.022 (2)
C7	0.0489 (18)	0.0562 (18)	0.106 (3)	0.0071 (15)	0.0148 (17)	−0.0217 (19)
C8	0.0460 (16)	0.0449 (15)	0.0490 (16)	0.0064 (13)	0.0061 (13)	0.0026 (13)
C9	0.0434 (15)	0.0456 (15)	0.0471 (16)	0.0044 (13)	0.0131 (13)	0.0009 (13)
C10	0.0455 (16)	0.0438 (14)	0.0474 (16)	−0.0001 (13)	0.0149 (13)	0.0013 (13)
C11	0.0478 (18)	0.0486 (16)	0.085 (2)	−0.0010 (14)	0.0170 (16)	−0.0034 (17)
C12	0.0443 (18)	0.069 (2)	0.096 (3)	−0.0064 (16)	0.0091 (17)	−0.006 (2)
C13	0.068 (2)	0.066 (2)	0.081 (2)	−0.0139 (18)	0.0166 (19)	−0.0177 (18)
C14	0.065 (2)	0.0560 (18)	0.078 (2)	0.0044 (16)	0.0167 (18)	−0.0150 (17)
C15	0.0504 (17)	0.0555 (17)	0.0610 (19)	0.0064 (14)	0.0148 (15)	−0.0089 (15)
C16	0.0435 (16)	0.0386 (14)	0.0478 (16)	0.0057 (12)	0.0087 (13)	−0.0025 (13)
C17	0.0494 (18)	0.0600 (18)	0.0606 (18)	0.0050 (14)	0.0145 (15)	0.0111 (15)
C18	0.0477 (19)	0.075 (2)	0.074 (2)	−0.0001 (16)	0.0154 (16)	0.0044 (19)
C19	0.0477 (19)	0.065 (2)	0.078 (2)	0.0080 (16)	−0.0017 (17)	−0.0048 (18)
C20	0.067 (2)	0.066 (2)	0.060 (2)	0.0142 (17)	−0.0012 (17)	0.0090 (17)
C21	0.0591 (19)	0.0528 (16)	0.0591 (18)	0.0061 (15)	0.0148 (15)	0.0070 (15)

### Geometric parameters (Å, °)

**Table:**

S1—C8	1.747 (3)	C10—C11	1.388 (4)
S1—C7	1.810 (3)	C10—C15	1.391 (3)
S2—C8	1.643 (3)	C11—C12	1.371 (4)
N1—C8	1.348 (3)	C11—H11	0.9300
N1—N2	1.375 (3)	C12—C13	1.383 (4)
N1—H1	0.8600	C12—H12	0.9300
N2—C9	1.293 (3)	C13—C14	1.360 (4)
C1—C2	1.3900	C13—H13	0.9300
C1—C6	1.3900	C14—C15	1.381 (4)
C1—C7	1.501 (3)	C14—H14	0.9300
C2—C3	1.3900	C15—H15	0.9300
C2—H2	0.9300	C16—C17	1.381 (4)
C3—C4	1.3900	C16—C21	1.386 (3)
C3—H3	0.9300	C17—C18	1.377 (4)
C4—C5	1.3900	C17—H17	0.9300
C4—H4	0.9300	C18—C19	1.355 (4)
C5—C6	1.3900	C18—H18	0.9300
C5—H5	0.9300	C19—C20	1.373 (4)
C6—H6	0.9300	C19—H19	0.9300
C7—H7A	0.9700	C20—C21	1.385 (4)
C7—H7B	0.9700	C20—H20	0.9300
C9—C10	1.472 (3)	C21—H21	0.9300
C9—C16	1.491 (3)		
			
C8—S1—C7	101.19 (13)	C11—C10—C9	121.0 (2)
C8—N1—N2	120.4 (2)	C15—C10—C9	121.1 (2)
C8—N1—H1	119.8	C12—C11—C10	121.3 (3)
N2—N1—H1	119.8	C12—C11—H11	119.4
C9—N2—N1	117.6 (2)	C10—C11—H11	119.4
C2—C1—C6	120.0	C11—C12—C13	119.8 (3)
C2—C1—C7	120.0 (2)	C11—C12—H12	120.1
C6—C1—C7	120.0 (2)	C13—C12—H12	120.1
C3—C2—C1	120.0	C14—C13—C12	119.9 (3)
C3—C2—H2	120.0	C14—C13—H13	120.0
C1—C2—H2	120.0	C12—C13—H13	120.0
C2—C3—C4	120.0	C13—C14—C15	120.5 (3)
C2—C3—H3	120.0	C13—C14—H14	119.7
C4—C3—H3	120.0	C15—C14—H14	119.7
C5—C4—C3	120.0	C14—C15—C10	120.6 (3)
C5—C4—H4	120.0	C14—C15—H15	119.7
C3—C4—H4	120.0	C10—C15—H15	119.7
C4—C5—C6	120.0	C17—C16—C21	118.4 (3)
C4—C5—H5	120.0	C17—C16—C9	121.1 (2)
C6—C5—H5	120.0	C21—C16—C9	120.5 (2)
C5—C6—C1	120.0	C18—C17—C16	120.9 (3)
C5—C6—H6	120.0	C18—C17—H17	119.5
C1—C6—H6	120.0	C16—C17—H17	119.5
C1—C7—S1	107.18 (18)	C19—C18—C17	120.2 (3)
C1—C7—H7A	110.3	C19—C18—H18	119.9
S1—C7—H7A	110.3	C17—C18—H18	119.9
C1—C7—H7B	110.3	C18—C19—C20	120.1 (3)
S1—C7—H7B	110.3	C18—C19—H19	119.9
H7A—C7—H7B	108.5	C20—C19—H19	119.9
N1—C8—S2	121.0 (2)	C19—C20—C21	120.1 (3)
N1—C8—S1	112.58 (19)	C19—C20—H20	119.9
S2—C8—S1	126.42 (15)	C21—C20—H20	119.9
N2—C9—C10	116.3 (2)	C20—C21—C16	120.2 (3)
N2—C9—C16	122.8 (2)	C20—C21—H21	119.9
C10—C9—C16	120.9 (2)	C16—C21—H21	119.9
C11—C10—C15	117.9 (3)		
			
C8—N1—N2—C9	170.5 (2)	C16—C9—C10—C15	3.9 (4)
C6—C1—C2—C3	0.0	C15—C10—C11—C12	1.1 (4)
C7—C1—C2—C3	179.7 (2)	C9—C10—C11—C12	−176.6 (3)
C1—C2—C3—C4	0.0	C10—C11—C12—C13	−0.7 (5)
C2—C3—C4—C5	0.0	C11—C12—C13—C14	−0.4 (5)
C3—C4—C5—C6	0.0	C12—C13—C14—C15	1.0 (5)
C4—C5—C6—C1	0.0	C13—C14—C15—C10	−0.6 (5)
C2—C1—C6—C5	0.0	C11—C10—C15—C14	−0.5 (4)
C7—C1—C6—C5	−179.7 (2)	C9—C10—C15—C14	177.3 (2)
C2—C1—C7—S1	−83.8 (2)	N2—C9—C16—C17	70.2 (3)
C6—C1—C7—S1	95.8 (2)	C10—C9—C16—C17	−109.1 (3)
C8—S1—C7—C1	164.8 (2)	N2—C9—C16—C21	−107.4 (3)
N2—N1—C8—S2	−179.69 (18)	C10—C9—C16—C21	73.4 (3)
N2—N1—C8—S1	−1.2 (3)	C21—C16—C17—C18	−1.2 (4)
C7—S1—C8—N1	−175.2 (2)	C9—C16—C17—C18	−178.8 (3)
C7—S1—C8—S2	3.2 (2)	C16—C17—C18—C19	1.1 (4)
N1—N2—C9—C10	179.0 (2)	C17—C18—C19—C20	−0.1 (5)
N1—N2—C9—C16	−0.3 (4)	C18—C19—C20—C21	−0.7 (4)
N2—C9—C10—C11	2.3 (4)	C19—C20—C21—C16	0.6 (4)
C16—C9—C10—C11	−178.4 (2)	C17—C16—C21—C20	0.3 (4)
N2—C9—C10—C15	−175.4 (2)	C9—C16—C21—C20	178.0 (2)

## References

[bb1] Ali, M. A., Baker, H. J. H. A., Mirza, A. H., Smith, S. J., Gahan, L. R. & Bernhardt, P. V. (2008). *Polyhedron*, **27**, 71–79.

[bb2] Ali, M. A., Mirza, A. H., Butcher, R. J., Tarafder, M. T. H., Keat, T. B., Ali, A. M. & Manaf, A. (2002). *J. Inorg. Biochem.* **92**, 141–148. 10.1016/s0162-0134(02)00559-7 12433421

[bb3] Allen, F. H., Kennard, O., Watson, D. G., Brammer, L., Orpen, A. G. & Taylor, R. (1987). *J. Chem. Soc. Perkin Trans. 2*, pp. S1–19.

[bb4] Bruker (2005). *APEX2*, *SAINT* and *SADABS* Bruker AXS Inc., Madison, Wisconsin, USA.

[bb5] Crouse, K. A., Chew, K.-B., Tarafder, M. T. H., Kasbollah, A., Ali, A. M., Yamin, B. M. & Fun, H.-K. (2004). *Polyhedron*, **23**, 161–168.

[bb6] Sheldrick, G. M. (2008). *Acta Cryst.* A**64**, 112–122. 10.1107/S0108767307043930 18156677

[bb7] Tarafder, M. T. H., Islam, M. T., Islam, M. A. A. A. A., Chantrapromma, S. & Fun, H.-K. (2008). *Acta Cryst.* E**64**, m416–m417. 10.1107/S1600536808002262 PMC2960465 21201362

[bb8] Tarafder, M. T. H., Kasbollah, A., Crouse, K. A., Ali, A. M., Yamin, B. M. & Fun, H.-K. (2001). *Polyhedron*, **20**, 2363–2370.

[bb9] Zheng, P.-W., Qiu, Q.-M., Lin, Y.-Y. & Liu, K.-F. (2006). *Acta Cryst.* E**62**, o1913–o1914.

